# Robot-assisted versus open kidney transplantation in patients who previously underwent an open kidney transplant

**DOI:** 10.1007/s00345-026-06754-x

**Published:** 2026-09-04

**Authors:** Luca Afferi, Isacco Donnini, Andrea Gallioli, Begonya Etcheverry, Joris Vangeneugden, Jeremy Mercier, Angelo Territo, Thomas Prudhomme, Lorenzo Masieri, Alessio Pecoraro, Natalia Ortiz Benitez, Rodrigo García-Baquero, Milla Ortved, Malene Rohrsted, Andreas Roder, Julia Abildgaard Dagnaes-Hansen, Francesc Vigués, Nicolas Doumerc, Joan Palou, Riccardo Campi, Sergio Serni, Karel Decaestecker, Alberto Breda

**Affiliations:** 1 Department of Surgery, Autonomous University of Barcelona, Barcelona, Spain; 2 Department of Urology, Fundació Puigvert, Barcelona, Spain; 3 Department of Urology, Austin Health, Melbourne, Australia; 4 Department of Urology, ASST Spedali Civili, University of Brescia, Brescia, Italy; 5 Department of Urology, Hospital Bellvitge, Bellvitge, Spain; 6 Department of Urology, Uz Gent, Gent, Belgium; 7 Department of Urology, Hospital of Toulouse, Toulouse, France; 8 Department of Experimental and Clinical Medicine, University of Florence-Unit of Oncology, Florence, Italy; 9 Unit of Urology and Renal Transplantation, University of Florence, Careggi Hospital, 50134 Florence, Italy; 10 Department of Urology, Puerta del Mar University Hospital, Cadiz, Spain; 11 Urological Research Unit, Department of Urology, Centre for Cancer and Organ Diseases, Copenhagen University Hospital-Rigshospitalet, Copenhagen, Denmark; 12 AZMM AZ Maria Middelares, Ghent, Belgium

**Keywords:** OKT, Open kidney transplantation, RAKT, Robot-assisted kidney transplantation, Living donors

## Abstract

**Introduction and objectives:**

The safety of robot-assisted kidney transplantation (RAKT) in patients with end-stage renal disease (ESRD) who underwent an open kidney transplantation (OKT) in the past is unknown. We tested the hypothesis that intraoperative and postoperative complication rates are similar between RAKT and OKT in this patient population.

**Materials and methods:**

This was a multicenter retrospective cohort study including 78 patients with ESRD who received RAKT or OKT from living donor between 2015 and 2023 in 7 European academic centers. All patients had received an OKT in the past. The primary outcome was to assess perioperative safety in terms of intra- and postoperative complications, described using the Clavien-Dindo classification. Secondary outcomes included dialysis-free survival (DFS), surgical reintervention-free survival (RFS) and renal function at discharge. Kaplan Meier estimates and log-rank test were used to compare DFS and RFS according to the surgical approach.

**Results:**

Among 78 patients who previously underwent OKT, 29 (37%) patients subsequently underwent RAKT and 49 (63%) OKT. Patients’ baseline characteristics were comparable between groups. The site of transplantation was the left iliac fossa in 24 (83%) and 30 (61%) patients undergoing RAKT and OKT, respectively (*p* = 0.07). Cold ischemia time was shorter in the RAKT group (60 vs. 114 min, *p* = 0.005), as was total vascular anastomosis time (33 vs. 38 min, *p* = 0.01). Estimated blood loss, rewarming time and intraoperative complications were similar in both groups. Early and late postoperative complications were similar in RAKT and OKT (respectively: 17% vs. 33%, *p* = 0.1; and 14% vs. 10%, *p* = 0.7). Over a median follow-up of 42 (IQR: 20–74) months, DFS was similar between RAKT and OKT, whereas RFS was higher in the RAKT group (100% vs. 84%, *p* = 0.02). Main limitations are represented by patient selection and the small sample size.

**Conclusions:**

In selected patients undergoing kidney re-transplantation after a previous OKT, RAKT appears to be technically feasible and provides perioperative outcomes comparable to the open approach. In this challenging surgical setting, RAKT may represent a valuable option in experienced centres.

## Introduction

 Kidney transplantation represents the gold standard for patients with end-stage renal disease (ESRD), providing clear benefits in terms of survival and quality of life compared to dialysis [1]. Robot-assisted kidney transplantation (RAKT) has been progressively adopted in selected centres since its introduction in 2015, demonstrating excellent results in terms of safety and reproducibility [2–4]. International multicentre experiences have demonstrated the feasibility of RAKT in several challenging scenarios, with clinical outcomes comparable to the conventional open approach [5, 6]. Furthermore, the use of modern techniques, such as intraoperative image superimposition, has allowed to spread the indication of RAKT even in patients with iliac artery calcification [7, 8].

However, there is still lack of evidence regarding the application of RAKT in patients already undergoing open kidney transplantation (OKT). In this scenario, the presence of a graft in the contralateral iliac fossa may adversely affect the performance of the procedure, particularly regarding trocar placement. Nevertheless, the possibility of reducing surgical morbidity in this population justifies the exploration of the feasibility of RAKT in this setting. The present study aims to compare RAKT and OKT in patients with history of a previous OKT in terms of peri and postoperative outcomes.

## Patients and methods

### Study design and setting

This retrospective observational study included patients with ESRD who had already undergone a previous OKT and subsequently received a second transplant, OKT or RAKT, from living donor between January 2015 and December 2023. Data were collected at seven European high-volume transplant centres within the ERUS-RAKT network.

### Inclusion and exclusion criteria

Inclusion criteria were age ≥ 18 years, a previous OKT, and clinical indication for re-transplantation. Patients with orthotopic transplants, extensive calcifications of the iliac vessels documented on a preoperative CT scan, active oncological diseases, uncontrolled systemic infections and severe comorbidities (cardiac, pulmonary, hepatic) were excluded. Finally, recipients of grafts with highly complex vascular anatomy (including more than 3 arteries, more than 1 small accessory artery, or more than 2 veins) were excluded from receiving RAKT.

### Donor and recipient data

The transplanted kidneys were exclusively from living donors. The data collected for each patient included age, sex, body mass index (BMI), the American Society of Anesthesiologists (ASA) score, Charlson Comorbidity Index (CCI), dialysis type (pre-emptive, haemodialysis, peritoneal), blood group compatibility, transplant site, laterality of the transplanted kidney, history of abdominal surgery, aetiology of ESRD and smoking status. For donors, age, sex and preoperative eGFR were documented.

During surgery, the following data were collected: duration of surgery, warm ischemia time (WIT), cold ischemia time (CIT), rewarming time (RT) and reperfusion times, duration of vascular anastomoses, anatomy of the graft, implantation site (right or left iliac fossa), uretero-vesical anastomosis type, estimated blood loss, any intraoperative complications, need for surgical conversion and drain placement. WIT was defined as the interval between donor renal artery clamping and the start of cold perfusion. CIT was defined as the interval between the start of cold perfusion and removal of the graft from cold storage. RT was defined as the interval between removal of the graft from cold storage and arterial reperfusion during implantation.

### Surgical techniques

All RAKT patients were operated with the da Vinci^®^ robotic system (Si, X or Xi), following the technical principles based on the Vattikuti-Medanta technique [9–13]. OKT patients were treated with standard open technique, with extraperitoneal access and anastomosis to the external iliac vessels [14]. In both groups, a double-J ureteral stent was placed. Intraabdominal drainage was placed selectively, based on the individual surgeon’s intraoperative assessment.

### Postoperative data

Postoperative complications were assessed up to 90 days after surgery and graded according to the Clavien-Dindo classification (CDC) [15]. Length of hospital stay, time to drain and double-J stent removal, need for renal biopsy, incidence of delayed graft function (DGF) and acute rejection episodes were also recorded.

### Outcomes

The primary outcome of the study was to assess perioperative safety in terms of intra- and postoperative complications, expressed with the CDC. Secondary outcomes included dialysis-free survival (DFS), surgical reintervention-free survival (RFS) and renal function at discharge, expressed as eGFR. RFS was defined as the time from transplantation to the first transplant-related reintervention. A reintervention was defined as any unplanned invasive procedure performed to diagnose or treat a post-transplant complication involving the renal graft, graft vasculature, or urinary tract. This included any unplanned surgical procedures requiring return to the operating room (e.g., bleeding control, vascular revision, graft repositioning), interventional radiology procedures (e.g., embolization, stent placement, percutaneous drainage), and endourological procedures (e.g., percutaneous nephrostomy, ureteral stent placement). Elective procedures, protocol biopsies, and planned staged procedures were not considered reinterventions. Reinterventions were identified through retrospective review of medical records at each participating center using the aforementioned criteria.

### Statistical analysis

Categorical variables were reported as numbers and percentages, with percentages rounded to the nearest integer. Continuous variables were reported as medians with interquartile ranges (IQR) and rounded to the nearest integer. Missing data were reported where present, and analyses were performed using complete-case analysis. Categorical variables in the RAKT and OKT groups were compared using Chi-square tests or Fisher’s exact tests. Continuous variables were compared using independent samples t-tests or Wilcoxon rank-sum tests, depending on whether their distribution was normal or non-normal. DFS and RFS were analysed using Kaplan-Meier survival curves and compared between groups with the log-rank test. All reported *P* values are two sided; a *P* value < 0.05 was considered statistically significant. Analyses were performed with R software (version 4.5.2, R Foundation for Statistical Computing, Vienna, Austria, 2020).

## Results

### Baseline characteristics

A total of 78 patients underwent kidney transplantation from a living donor, of which 49 (63%) underwent OKT and 29 (37%) underwent RAKT. Baseline characteristics are presented in Table 1. The median recipient age was 49 years, and 51% of patients were male in both groups. An age-unadjusted CCI of 0–2 was observed in 35 (71%) patients in the OKT and 22 (76%) patients in the RAKT group (*p* = 0.5). The etiology of kidney insufficiency differed significantly between groups (*p* = 0.007): glomerulonephritis was most prevalent in the OKT group (35%), whereas both glomerulonephritis and IgA nephropathy were most common in the RAKT group (each 24%). No statistically significant differences were observed between groups with respect to ASA score, BMI, smoking status, type of dialysis, or AB0 compatibility. Additionally, donor characteristics did not differ significantly between the two groups.

### Intraoperative data

Intraoperative data are reported in Table 2. The implant site was most frequently the left iliac fossa in both groups (83% for RAKT vs. 61% for OKT; *p* = 0.07). Operative time was similar between RAKT and OKT (202 vs. 192 min; *p* = 0.3). The median CIT and total time for vascular anastomosis were shorter in the RAKT group (respectively: 60 vs. 114 min, *p* = 0.005; and 33 vs. 38 min, *p* = 0.01). Estimated blood loss, RT, and intraoperative complications were not significantly different between groups. No conversion from RAKT to open surgery was reported.

### Postoperative data

Perioperative results are reported in Table 3. Median length of hospital stay was similar in both groups (8 days; *p* = 0.1). Median time to drain removal was similar between OKT and RAKT (6 vs. 4 days; *p* = 0.4), while removal of the double-J ureteral stent occurred later in RAKT patients (28 vs. 34 days; *p* = 0.001). There was no statistically significant difference in the incidence of DGF between groups (3% RAKT vs. 10% OKT; *p* = 0.4).

### Postoperative complications

Data about postoperative complications is reported in Table 4. Patients with postoperative complications were 8 (28%) in the RAKT group and 17 (35%) in the OKT group (*p* = 0.6). Early postoperative complications (within 30 days) were observed in 5 (17%) RAKT and 16 (33%) OKT patients (*p* = 0.1), whereas late postoperative complications (31–90 days) occurred in 4 (14%) and 5 (10%) patients, respectively (*p* = 0.7).There was no statistically significant difference in major complications (CDC ≥ III) between the groups.

### Follow-up

**Fig. 1:**
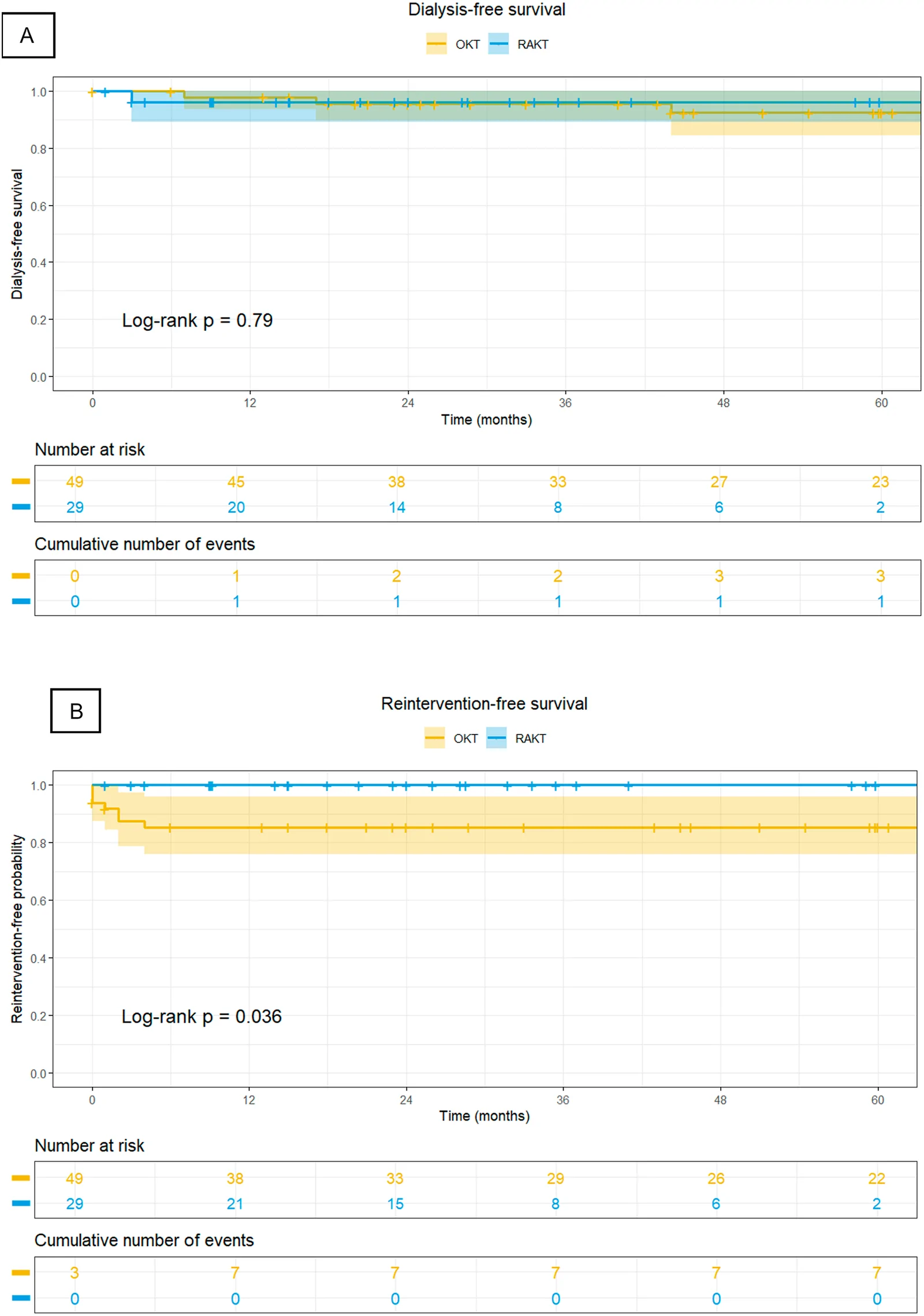
Kaplan-meier curves estimating **A** dialysis-free survival and **B** reintervention-free survival in 78 patients undergoing open kidney transplantation (OKT) or robot-assisted kidney transplantation (RAKT) after a previous OKT

**Fig. 2:**
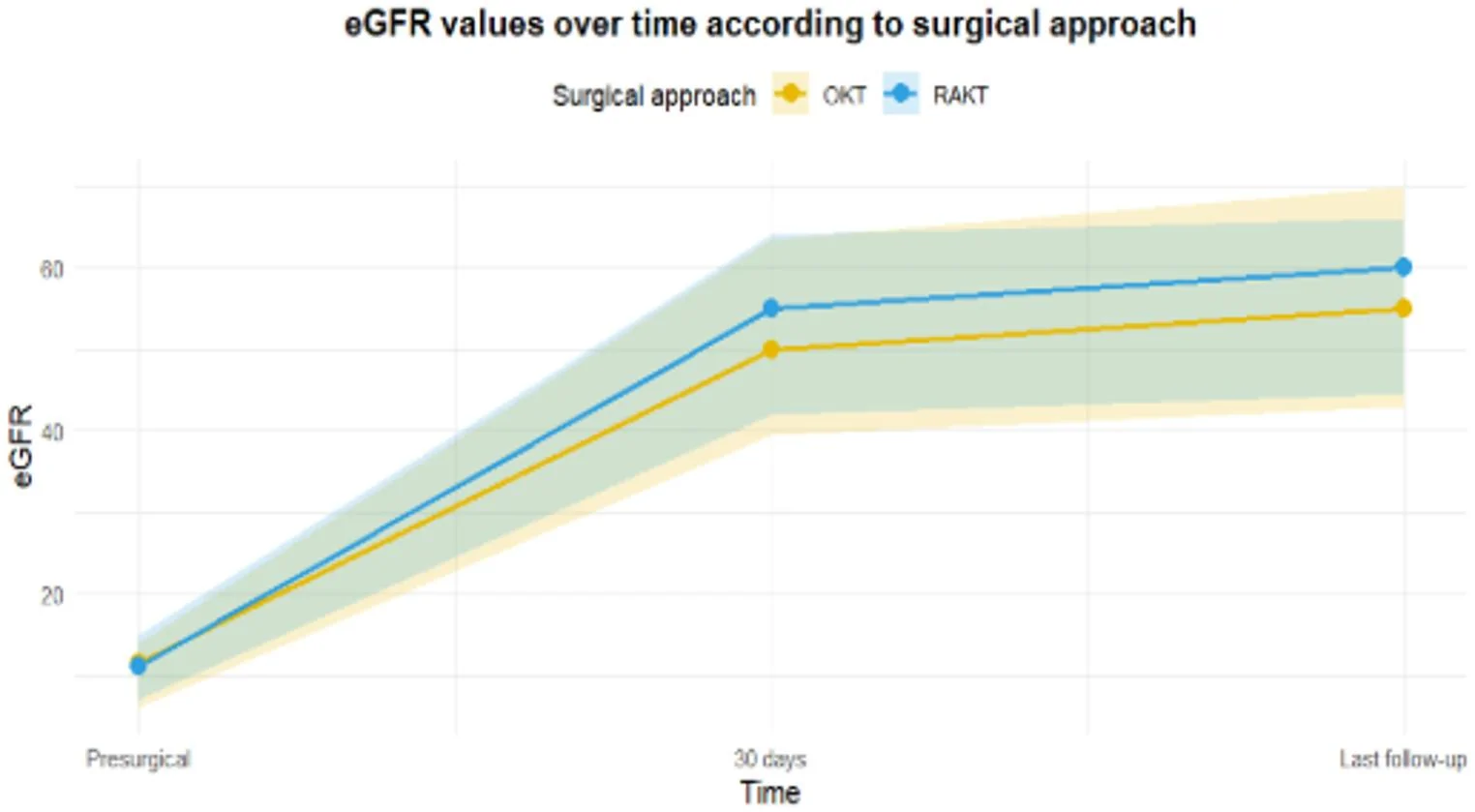
Estimated glomerular filtration rate (eGFR) levels in 78 patients undergoing open kidney transplantation (OKT) or robot-assisted kidney transplantation (RAKT) after a previous OKT

Over a median follow-up of 42 (IQR: 20–74) months, DFS was similar between groups, whereas RFS was better in the RAKT group (100% vs. 84%; *p* = 0.02), as shown in Fig. 1. A total of eight reinterventions were recorded, all of which were in the OKT group. The reasons for reintervention consisted of renal artery stenosis requiring stent placement (*n* = 2), fascial dehiscence necessitating resuturing in the operating room (*n* = 1), compromised graft perfusion due to renal vein kinking (*n* = 1), postoperative hematoma requiring percutaneous drainage (*n* = 1), active bleeding of the inferior epigastric artery requiring embolization (*n* = 1), and hydronephrosis secondary to ureteral stenosis requiring percutaneous nephrostomy (*n* = 2). Renal function at 30 days, measured by eGFR, was similar between the two groups (Fig. 2).

## Discussion

The possibility of offering a second kidney transplant in patients who already underwent OKT represents a complex clinical and surgical challenge. OKT remains the gold standard in this setting [16]. The adoption of the robotic technique in such patients, even though reported in some studies, has not been explored in detail to date.

This multicentre study represents the first comparative analysis between RAKT and OKT in patients undergoing kidney transplantation after a previous OKT. Our findings show that both functional and perioperative outcomes were comparable between the two surgical approaches. Specifically, no differences were observed in the incidence of DGF or in eGFR at 30 days after transplantation, nor in the rates of overall or high-grade perioperative complications. There were no cases of conversion from RAKT to open surgery, and the intraoperative complication rate remained low, in line with what has been observed in other European reports [13, 17]. These findings suggest that the robotic approach is a feasible and safe option in experienced hands for patients with a prior OKT, consistent with previous reports in both standard and complex transplant settings, including the use of grafts with multiple vessels and orthotopic transplantation [7, 18, 19].

In our cohort, no statistically significant differences in perioperative complications were observed between the OKT and RAKT groups. Although numerically more postoperative complications were recorded in the OKT group, this difference was largely driven by non-surgical complications, such as pneumonia or cardiac angina, which are unlikely to be directly related to the surgical approach itself. Given the limited sample size and retrospective design, these findings should be interpreted cautiously, as selection bias and unmeasured confounding may have influenced the observed distribution of complications. Nevertheless, the rate of high-grade surgical complications was low and comparable in both groups, in line with recent reports [20, 21].

All reinterventions in our series occurred in the OKT cohort and were mainly related to vascular and urological complications. However, given the limited sample size this observation should be interpreted cautiously. Differences in patient selection and potential heterogeneity in surgical experience across centres may also have contributed to these findings. Overall, these results suggest that RAKT provides perioperative outcomes at least comparable to OKT in this technically challenging clinical scenario, supporting its feasibility in appropriately selected patients.

Interestingly, CIT was shorter in the RAKT group compared with the OKT group, as reported also in previous studies [21]. This finding may be explained by logistical and procedural factors rather than the surgical technique itself. In many centres, RAKT procedures are carefully scheduled with the recipient surgical bed already prepared in the operating room at the time of bench surgery, allowing implantation to proceed without delay. In contrast, in the open approach, the graft may occasionally arrive before the recipient procedure is fully ready to proceed, resulting in longer CIT. Additionally, RAKT programs are often implemented in highly specialised centres with well-coordinated surgical teams and streamlined workflows, which may further contribute to minimizing CIT.

The RT was slightly longer in the RAKT group, likely reflecting the additional time required for robotic docking and trocar placement. Conversely, the RAKT group showed a shorter total vascular anastomosis time compared with OKT. This reduction may reflect the precision and stable visualization provided by the robotic platform, as well as the different technique used for robotic vascular suturing. Moreover, the high level of experience of the robotic surgical teams, compared with the potentially more heterogeneous surgical teams involved in OKT, may have contributed to a faster anastomotic performance in this study. However, we believe that this modest difference in vascular anastomosis time, although statistically significant, is unlikely to translate into clinically meaningful differences in perioperative outcomes.

Most patients in our cohort received a surgical drain intraoperatively, which was removed shortly after surgery in both groups. The decision to place a drain was primarily based on surgeon preference, and the present study does not allow any conclusions regarding its potential benefits in this setting. Importantly, practice patterns regarding drain placement vary widely across transplant programs, and some centres performing kidney transplantation do not routinely use surgical drains [22, 23]. Regarding double-J stent placement, this was adopted in all cases in our cohort. However, other experiences worldwide suggest that ureteral stenting may be used selectively [24]. Current guidelines do not define a specific timing for double-J stent removal. Nevertheless, meta-analyses conducted in the setting of OKT have shown that early double-J stent removal may be associated with a lower risk of infectious complications without increasing the rate of urological complications [16, 25, 26]. Comparable evidence is currently limited for RAKT. In our series, surgeons likely adopted a more cautious approach to the ureterovesical anastomosis by maintaining the stent for a slightly longer period.

Several limitations must be considered when interpreting the results. Firstly, the retrospective, non-randomised design of the study carries an inherent risk of selection bias. Given the observational nature of the study, unmeasured variables may have influenced treatment allocation and perioperative outcomes. While this represents the largest multicentre series published to date on this topic, the small sample size limits the statistical power to detect significant differences in outcomes between the two approaches and increases the risk of type II error. Furthermore, centre- and surgeon-specific effects were not formally analysed or adjusted for in the present study. In particular, information regarding the individual surgeons performing the procedures was not systematically reported. While RAKT was performed by surgeons with substantial experience in both OKT and robotic surgery, procedures in the OKT group were likely performed by surgeons with varying levels of experience across centres. This heterogeneity in surgical expertise may have influenced perioperative outcomes, including complication rates and operative times. In addition, potential learning-curve effects associated with the adoption of RAKT could not be assessed in the present analysis. Differences in institutional experience and procedural volume across centres may therefore have influenced the observed outcomes. Finally, the findings of this study primarily reflect outcomes in highly selected patients treated at centres with established expertise in kidney transplantation and robotic surgery, which may limit the generalizability of these results to other settings.

## Conclusions

In patients undergoing heterotopic kidney transplantation after a previous OKT, RAKT proves to be technically feasible and to be associated with a safety profile comparable to OKT. In a scenario where re-transplantation might represent a surgical challenge, robotic surgery could be a strategic option to be exploited. Prospective cohort studies and cost-benefit analyses will be crucial not only to confirm these results, but also to define the role of RAKT in complex transplantation pathways.

**Table 1:** Baseline characteristics of 78 patients receiving open kidney transplantation (OKT) or robot-assisted kidney transplantation (RAKT) from living donor after a previous OKT

Variables	OKT (*N* = 49, 63%)	RAKT (*N* = 29, 37%)	*p*-value
Recipients			
Age [years], median (IQR)	49 (43–56)	49 (41–60)	0.5
Male sex, n (%)	25 (51)	15 (51)	1
ASA score, n (%)			
1–2	4 (8)	7 (24)	0.1
3–4	45 (92)	22 (76)	
CCI (age unadjusted), n (%)			
0–2	35 (71)	22 (76)	0.5
3–5	14 (29)	6 (21)	
Missing	0	1 (3)	
BMI [kg/m^2^], median (IQR)	24 (22–26)	25 (22–29)	0.5
Smoking status, n (%)			
Never smoker	32 (65)	19 (66)	0.4
Previous smoker	12 (25)	3 (10)	
Active smoker	5 (10)	2 (7)	
Missing	0	5 (17)	
Type of dialysis, n (%)			
No dialysis (preemptive)	16 (33)	13 (45)	0.4
Hemodialysis	26 (53)	14 (48)	
Peritoneal dialysis	7 (14)	2 (7)	
Aetiology of ESRD, n (%)			
Diabetes mellitus	1 (2)	0	***0.007***
Glomerulonephritis	17 (35)	7 (24)	
ADPKD	0	2 (7)	
Interstitial nephritis	8 (16)	2 (7)	
IgA nephropathy	1 (2)	7 (24)	
Obstructive uropathy	1 (2)	0	
Others*	21 (43)	11 (38)	
AB0 compatibility, n (%)	37 (75)	19 (65)	0.1
Missing	3 (6)	0	
Donors			
Donor age, median (IQR)	52 (43–59)	53 (45–62)	0.4
Donor eGFR, median (IQR)	97 (88–100)	100 (90–105)	0.5

*ASA* American Society of anesthesiologists, *CCI* Charlson Comorbidity Index, *ESRD* End-stage renal disease, *ADPKD* Autosomal dominant polycystic kidney disease, *eGFR* estimated glomerular filtration rate (ml/min/1.73m^2^)

*Includes unknown and amyloidosis

**Table 2:** Surgical characteristics of 78 patients undergoing open kidney transplantation (OKT) or robot-assisted kidney transplantation (RAKT) from living donor after a previous OKT

Variables	OKT (*N* = 49, 63%)	RAKT (*N* = 29, 37%)	*P*-value
Bench surgery			
Donor kidney side (left), n (%)	25 (51)	25 (86)	***0.01***
Vascular anatomy, n (%)			
1 artery, 1 vein	31 (63)	23 (80)	0.08
≥1 arteries, 1 vein	15 (31)	3 (10)	
Missing	3 (6)	3 (10)	
Ureteral anomalies, n (%)	0	1 (3)	1
Kidney transplantation			
Transplantation site, n (%)			
Left iliac fossa	30 (61)	24 (83)	0.07
Right iliac fossa	19 (39)	5 (17)	
Type of incision, n (%)			
Gibson	23 (47)	0	***< 0.001***
Pararectal	22 (45)	0	
Periumbelical incision	0	12 (41)	
Pfannestiel incision	0	12 (41)	
Midline	0	5 (18)	
Other	4 (8)	0	
Surgical time [min], median (IQR)	192 (130–241)	202 (145–250)	0.3
Warm ischemia time [min], median (IQR)	3 (2–4)	2 (2–3)	0.2
Cold ischemia time [min], median (IQR)	114 (82–183)	60 (35–120)	***0.005***
Rewarming time [min], median (IQR)	38 (33–45)	42 (38–55)	0.06
Total time for the vascular anastomosis [min], median (IQR)	38 (33–47)	33 (26–40)	***0.01***
Estimated blood loss [ml], median (IQR)	207 (100–300)	200 (100–300)	0.7
Intraoperative complications, n (%)	1 (2)	2 (6)	0.5
Drainage placement, n (%)	31 (63)	19 (65)	0.7

**Table 3:** Perioperative data of 78 patients undergoing open kidney transplantation (OKT) or robot-assisted kidney transplantation (RAKT) from living donor after a previous OKT

Variable	OKT (*N* = 49, 63%)	RAKT (*N* = 29, 37%)	*P*-value
Length of hospital stay [days], median (IQR)	8 (7–11)	8 (7–9)	0.1
Days to drain removal, median (IQR)	6 (4–8)	4 (3–5)	0.4
Days to double-J stent removal, median (IQR)	28 (21–33)	34 (24–45)	***0.001***
Delayed graft function, n (%)	5 (10)	1 (3)	0.4
Postoperative graft biopsy, n (%)	7 (14)	6 (21)	1
Postoperative complications			
Patients with postoperative complications, n (%)	17 (35)	8 (28)	0.6
Early* postoperative complications, n (%)	16 (33)	5 (17)	0.1
Early* postoperative complications CDC ≥ 3, n (%)	9 (18)	1 (3)	0.07
Late** postoperative complications, n (%)	5 (10)	4 (14)	0.7
Late** postoperative complications CDC ≥ 3, n (%)	2 (4)	3 (10)	0.3

*CDC* Clavien Dindo Classification

*Early = within 30 days

**Late = 31–90 days

**Table 4:** Early postoperative complications in patients undergoing open kidney transplantation (OKT) or robot-assisted kidney transplantation (RAKT) from living donor after a previous OKT

Type of complication	Early postoperative complications (within 30 days) OKT *N* = 16|RAKT *N* = 5						Late postoperative complications (31–90 days) OKT *N* = 5|RAKT *N* = 4		
	Grade 1 & 2		Grade 3a & 3b		Grade 4a & 4b	Grade 5	Grade 1 & 2	Grade 3a & 3b	
	OKT	RAKT	OKT	RAKT	OKT	OKT	OKT	OKT	RAKT
Infective, n (%)									
Urinary tract infection	3 (20)	1 (20)					3 (60)		
Sepsis						1 (6)			
Cardiac, n (%)									
Myocardial infarction					1 (6)				
Cardiac angina			1 (6)						
Respiratory, n (%)									
Pneumonia	1 (6)								
Surgical, n (%)									
Wound dehiscence			1 (6)	1 (20)					
Bleeding/hematoma		1 (20)	2 (13)						
Ureteric stenosis			2 (13)					1 (20)	
Lymphocele									2 (50)
Vascular, n (%)									
Renal artery stenosis								1 (20)	
DVT/thromboembolic event	1 (6)								1 (25)
Venous kinking (kidney repositioning)			1 (6)						
Functional, n (%)									
Acute kidney injury		1 (20)							
Acute rejection (graft biopsy)	1 (6)								1 (25)
General/systemic, n (%)									
Anemia (transfusions)	1 (6)	1 (20)							

Denominator refers to total number of complications by technique and by postoperative period

*DVT* deep vein thrombosis

## Data Availability

The data supporting the findings of this study are not publicly available due to ethical and privacy restrictions but are available from the corresponding author upon reasonable request, subject to institutional and ethical approvals.

## Declarations

### Statement on the welfare of animals

This article does not contain any studies/experiments with human participants or animals performed by any of the authors.
